# Few-layer hexagonal bismuth telluride (Bi_2_Te_3_) nanoplates with high-performance UV-Vis photodetection†

**DOI:** 10.1039/d0na00006j

**Published:** 2020-02-10

**Authors:** Ye Zhang, Qi You, Weichun Huang, Lanping Hu, Jianfeng Ju, Yanqi Ge, Han Zhang

**Affiliations:** SZU-NUS Collaborative Innovation Centre for Optoelectronic Science & Technology, International Collaborative Laboratory of 2D Materials for Optoelectronics Science and Technology of Ministry of Education, Institute of Microscale Optoelectronics, Shenzhen University Shenzhen 518060 China hzhang@szu.edu.cn; Nantong Key Lab of Intelligent and New Energy Materials, College of Chemistry and Chemical Engineering, Nantong University Nantong 226019 Jiangsu P. R. China

## Abstract

It is widely known that the excellent intrinsic electronic and optoelectronic advantages of bismuthene and tellurene make them attractive for applications in transistors and logic and optoelectronic devices. However, their poor optoelectronic performances, such as photocurrent density and photoresponsivity, under ambient conditions severely hinder their practical application. To satisfy the demand of high-performance optoelectronic devices and topological insulators, bismuth telluride nanoplates (Bi_2_Te_3_ NPs) with different sizes, successfully synthesized by a solvothermal approach have been, for the first time, employed to fabricate a working electrode for photoelectrochemical (PEC)-type photodetection. It is demonstrated that the as-prepared Bi_2_Te_3_ NP-based photodetectors exhibit remarkably improved photocurrent density, enhanced photoresponsivity, and faster response time and recovery time in the UV-Vis region, compared to bismuthene and tellurene-based photodetectors. Additionally, the PEC stability measurements show that Bi_2_Te_3_ NPs have a comparable long-term stability for on/off switching behaviour for the bismuthene and tellurene-based photodetectors. Therefore, it is anticipated that the present work can provide fundamental acknowledgement of the optoelectronic performance of a PEC-type Bi_2_Te_3_ NP-based photodetector, shedding light on new designs of high-performance topological insulator-based optoelectronic devices.

## Abstract 2

Topological insulator bismuth telluride (Bi_2_Te_3_) nanoplates have been employed to remarkably improve photoelectrochemical performance.

## Graphical abstract

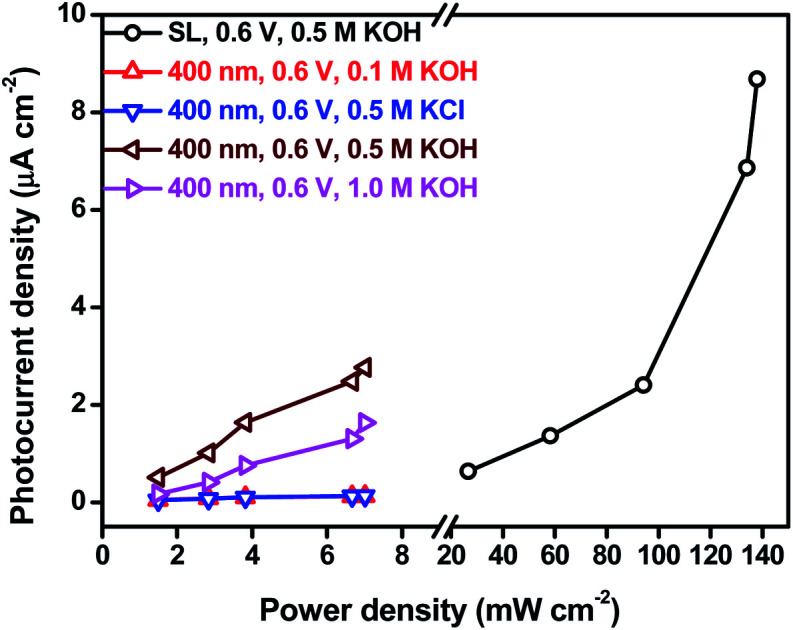

## Introduction

The individual elements tellurium (Te) and bismuth (Bi) have been widely applied to the fabrication and applications of photodetectors over the past decade due to their simple composition, excellent nonlinear photonic performance,^1–4^ and intriguing thermoelectric^5–7^ and photoelectric properties.^8–10^ Similar to the famous black phosphorus (BP), both Te and Bi have a layer-dependent energy band gap (*E*_g_)^1,3,8^ that can be easily tuned (Te: 0.35–1.0 eV;^1^ Bi: 0–0.55 eV (ref. 3)) when the thickness of Te or Bi decreases from bulk to monolayer. Recently, Ye *et al.* reported that ultrathin 2D nonlayered Te nanosheets synthesized by a substrate-free solution process, displayed high on/off ratios (10^6^), remarkable field-effect mobility (700 cm^2^ V^−1^ s^−1^) and comparable air-stable performance.^8^ Besides, the ultrathin Te nanosheets fabricated by a simple liquid phase exfoliation (LPE) method, showed excellent photoresponse behaviors from the UV to the visible region in association with strong time and cycle stability for the on/off switching behaviors.^10^ In 2017, Zhang *et al.* reported that ultrasmall Bi quantum dots fabricated by a LPE approach, exhibited good photoresponse performance from the UV to visible region as well as long-term photoresponse stability.^11^ Nevertheless, the poor photoresponse performances of Te or Bi nanomaterials, especially photoelectrochemical (PEC) photocurrent density and photoreponsivity, still severely limit their device development for practical applications. Therefore, it is still a challenge to explore a new method to improve their photoresponse performance under ambient conditions.

However, bismuth telluride (Bi_2_Te_3_), basically known as a compound of the post-transition metal element Bi and the non-metal element Te, also exhibits a thickness-dependent *E*_g_ (from 0.16 eV to 1.36 eV),^12,13^ and a high structural stability.^14,15^ Versatile strategies have already been employed to synthesize Bi_2_Te_3_ nanomaterials, including template synthesis,^16^ evaporation,^17^ electrochemical deposition,^18^ chemical solution process,^19,20^ solvothermal approaches^21,22^ and microwave-assisted methods.^23^ Bi_2_Te_3_ as one of the common topological insulators, features an unconventional phase of quantum matter possessing an insulating bulk state as well as a metallic surface state.^17^ Such metallic surface states were experimentally evidenced to be protected by time-reversal symmetry and demonstrated to be robust against non-magnetic perturbation.^24,25^ In addition, topological insulator Bi_2_Te_3_ exhibits an excellent surface mobility^26^ and good optoelectronic performance.^27^ This, combined with the relatively narrow *E*_g_ of Bi_2_Te_3_ and low cost and facile synthesis of Bi_2_Te_3_ nanomaterials, has drawn great interest in photodetection,^28–30^ field effect transistors,^26,31^ spintronics,^32,33^ thermoelectrics,^22,34^ and lasers.^35–37^ These advantages of Bi_2_Te_3_ merit it to be qualified for the practical application in high-performance optoelectronic devices.

In this work, Bi_2_Te_3_ nanoplates (NPs) with a rhombohedral phase in the space group *D*^5^_3d_(*R*3*m*), have been successfully synthesized by a solvothermal approach. To determine the size-dependent PEC performances of Bi_2_Te_3_ NPs, different sizes of Bi_2_Te_3_ NPs were readily obtained by simply tuning the reaction time. The as-synthesized Bi_2_Te_3_ NPs were, for the first time, developed as working materials to fabricate a PEC-type photodetector in various electrolytes. The PEC results demonstrate that the Bi_2_Te_3_ NP-based photodetectors exhibit not only a largely improved photocurrent density and photoresponsivity, but also a comparable photoresponse stability compared to that of Bi or Te nanomaterial-based photodetectors. It is anticipated that this work can provide fundamental guidance for constructing high-performance Bi_2_Te_3_ NP-based photodetectors, paving the way to new designs of topological insulator-based optoelectronic devices with excellent properties.

## Methods

### Materials

Poly(vinyl pyrrolidone) (PVP, K30), poly(vinylidene fluoride) (PVDF, *M*_w_ = 534 000 g mol^−1^), Bi(NO_3_)_3_·5H_2_O, dimethyl formamide (DMF, 99.9%), acetone, and ethanol were purchased from Sigma-Aldrich. Sodium tellurite (Na_2_TeO_3_, 99.9%) and indium tin oxide (ITO) were purchased from Aladdin Co., Inc. All chemical reagents were used without further purification. Double-distilled deionized (DI) water was used for synthesis.

### Synthesis of Bi_2_Te_3_ hexagonal NPs

In a typical procedure, 20 mmol Bi(NO_3_)_3_·5H_2_O and 40 mmol Na_2_TeO_3_ were first dissolved in 30 mL DI water. Then 0.03 mmol PVP was added into the solution and it was kept stirring for 30 min to form a homogeneous mixture. The mixture was transferred into a 50 mL Teflon-lined autoclave and placed in an oven at 180 °C. After a predetermined reaction time (2 h or 12 h), the reaction was stopped by quenching the system to room temperature. The Bi_2_Te_3_ hexagonal NPs were obtained by centrifugation at 4000 rpm for 20 min and washed with deionized water, ethanol and acetone, each. The product was finally dried in a vacuum oven at 80 °C overnight for the next use.

### Characterization

The morphologies and dimensions of Bi_2_Te_3_ NPs were determined by both scanning electron microscopy (SEM, Hitachi-SU8010) and transmission electron microscopy (TEM, FEI Tecnai G2 F30). High-resolution TEM (HRTEM) was performed to determine the atomic arrangements of the as-synthesized Bi_2_Te_3_ NPs. Energy-dispersive X-ray spectroscopy (EDS) was carried out using an FEI Tecnai G2 F30 TEM equipped with an Oxford EDAX EDS system. Atomic force microscopy (AFM, Bruker, with 512 pixels per line) was performed after depositing a drop of dispersion onto a silicon substrate. The X-ray diffraction (XRD) analysis was performed on an X'Pert-Pro MPD diffractometer with a Cu K-α radiation source at room temperature. Ultraviolet-visible (UV-Vis) absorption spectroscopy was performed with a spectral range of 200–1500 nm by using a UV-Vis absorbance spectrometer (Cary 60, Agilent) at room temperature.

### Photoresponse activity

The PEC measurement system in Scheme S1† was used to characterize the photoresponse behaviour of Bi_2_Te_3_ NPs. A standard three-electrode system, that is, a working electrode (for example, Bi_2_Te_3_ NPs deposited on ITO-coated glass, photoanode), a counter electrode (platinum wire, photocathode), and a reference electrode (Ag/AgCl electrode), was assembled in various aqueous electrolytes, including KOH (0.1 M, 0.5 M, and 1.0 M), KCl (0.5 M), and HCl (0.5 M). To ensure good adhesion between ITO-coated glass and the sample, the as-synthesized samples were first re-dispersed in a 0.2 mg mL^−1^ PVDF/DMF solution, and then the dispersion was deposited onto ITO-coated glass and dried under vacuum at 80 °C overnight. Electrochemical impedance spectra (EIS) were obtained in the frequency range from 1 to 10^5^ Hz with an amplitude of 0.005 V. Amperometric current–time (*I*–*t*) curves were recorded at bias voltages of 0 V, 0.3 V, and 0.6 V with increasing power densities at a sampling interval of 5 s. Simulated light (300–800 nm) and lasers with different *λ* (*λ* = 365 nm, 400 nm, 475 nm, 550 nm, 600 nm and 700 nm) were employed to irradiate the Bi_2_Te_3_ NP-based photodetectors. Light power densities (*P*_*λ*_) of these irradiations with labels of Dark, I, II, III, IV, and VI levels gradually increased (Table S1†). As a control experiment, a piece of naked ITO-coated glass was also irradiated by using a SL under the same conditions.

## Results and discussion

**Fig. 1:**
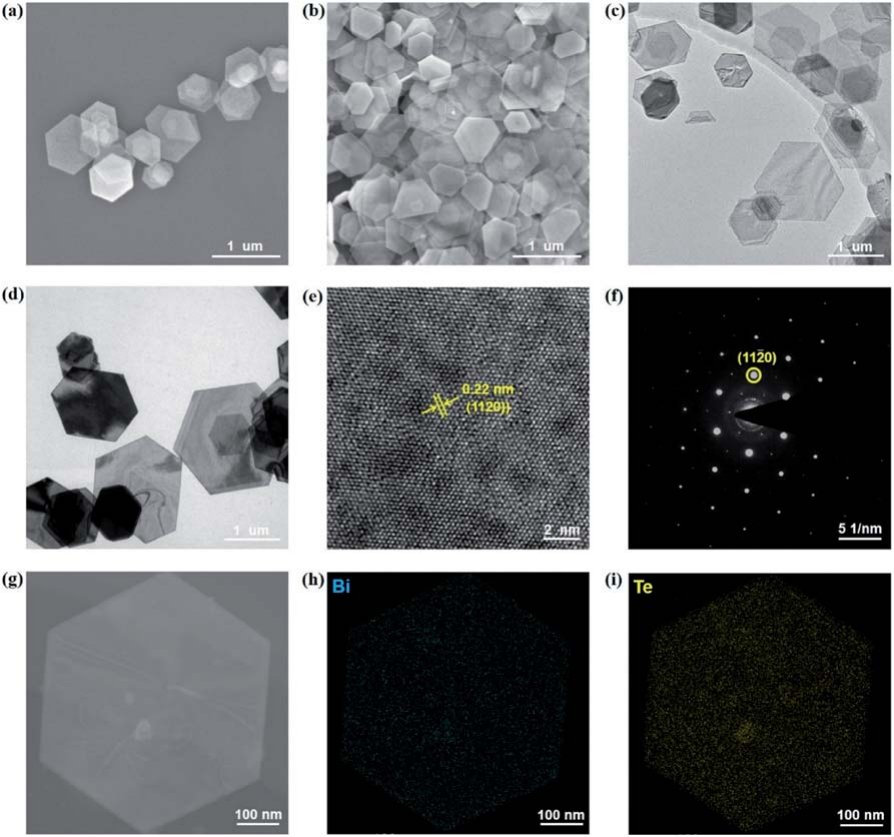
Structural characterization of the as-prepared Bi_2_Te_3_ NPs. SEM images of (a) Bi_2_Te_3_ NPs-1 and (b) Bi_2_Te_3_ NPs-2. TEM images of (c) Bi_2_Te_3_ NPs-1 and (d) Bi_2_Te_3_ NPs-2. (e) HRTEM image of Bi_2_Te_3_ NPs-1. (f) SAED pattern of Bi_2_Te_3_ NPs-1. (g) TEM image of one single hexagonal Bi_2_Te_3_ NP and results of its EDS elemental mapping of (h) Bi and (i) Te shown in (g).

The Bi_2_Te_3_ NPs with a well-defined hexagonal shape were synthesized by the solvothermal method. In order to investigate the influence of the size of Bi_2_Te_3_ NPs on photoresponse performances, two kinds of Bi_2_Te_3_ NPs with different sizes have been facilely synthesized by tuning the reaction time. For convenience, the Bi_2_Te_3_ NPs reacted after 2 h and 12 h are abbreviated as Bi_2_Te_3_ NPs-1 and Bi_2_Te_3_ NPs-2, respectively. Fig. 1a and b give the SEM images of the as-prepared Bi_2_Te_3_ NPs-1 and Bi_2_Te_3_ NPs-2, both of which exhibit a well-defined hexagonal shape, while the average lateral dimensions of Bi_2_Te_3_ NPs-1 and Bi_2_Te_3_ NPs-2 are 620 ± 150 nm and 730 ± 210 nm, respectively. The TEM characterization (Fig. 1c and d) reveals that the as-prepared Bi_2_Te_3_ NPs also represent a well-defined hexagonal shape. Besides, Bi_2_Te_3_ NPs-2 have darker hexagons (Fig. 1d), compared to Bi_2_Te_3_ NPs-1 (Fig. 1c), indicating that the longer the reaction time the thicker the Bi_2_Te_3_ NPs are, which is in good agreement with the “oriented attachment” mechanism of Bi_2_Te_3_ NPs.^38,39^ The HRTEM image (Fig. 1e) shows a clear lattice spacing of 0.22 nm, consistent with the (1120) plane of layered Bi_2_Te_3_.^27^ Sharp diffraction spots are observed in the selected area electron diffraction (SAED) pattern (Fig. 1f), and the EDS line scan analysis (Fig. 1g) reveals compositional variation in a single Bi_2_Te_3_ NP, suggesting that Bi and Te are evenly distributed (Fig. 1h and j).

**Fig. 2:**
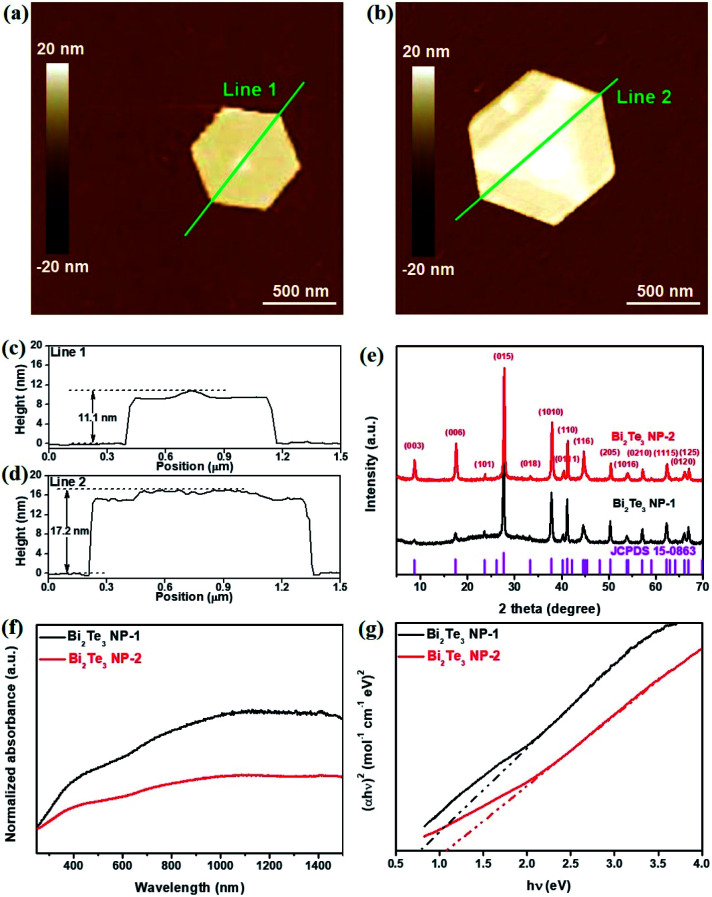
AFM images of (a) Bi_2_Te_3_ NPs-1 and (b) Bi_2_Te_3_ NPs-2. (c, d) Height profiles along the green lines in (a) and (b), respectively. (e) XRD patterns of Bi_2_Te_3_ NPs-1 and Bi_2_Te_3_ NPs-2. (f) UV-Vis spectra of the as-prepared Bi_2_Te_3_ NPs-1 and Bi_2_Te_3_ NPs-2 and (g) their Tauc plots for the calculations of *E*_g_.

The thicknesses of the as-synthesized Bi_2_Te_3_ NPs-1 and Bi_2_Te_3_ NPs-2 were characterized by AFM, as shown in Fig. 2a and b, respectively. It can be clearly seen in Fig. 2c and d that with the increase in the reaction time, the measured thickness of Bi_2_Te_3_ NPs obviously increases from 11.1 nm to 17.2 nm, which correspond to 11 and 17 layers, respectively, given that one layer is regarded as an average quintuple layer of Te–Bi–Te–Bi–Te with a thickness of 1.0 nm.^17^ XRD patterns of the as-synthesized Bi_2_Te_3_ NPs, as shown in Fig. 2e, can be indexed to a rhombohedral Bi_2_Te_3_ structure (JCPDS Card Number 15-0863). UV-Vis absorption spectroscopy was employed to characterize the optical response of differently sized Bi_2_Te_3_ NPs (Fig. 2f). Broadband absorption from 260 nm to 1500 nm, is observed for Bi_2_Te_3_ NPs-1 and Bi_2_Te_3_ NPs-2, which is in good agreement with previously reported results,^40,41^ implying great potential for application in broadband optoelectronic devices. Besides, Tauc plots of Bi_2_Te_3_ NPs-1 and Bi_2_Te_3_ NPs-2 (Fig. 1g) were calculated based on the results in Fig. 1f, and size-dependent *E*_g_ values of 0.83 eV (Bi_2_Te_3_ NPs-1) and 1.0 eV (Bi_2_Te_3_ NPs-2) were obtained, close to that of the previously reported Bi_2_Te_3_ nanoparticles,^42^ suggesting that the *E*_g_ of Bi_2_Te_3_ NPs could be easily controlled by simply tuning the reaction conditions.

**Fig. 3:**
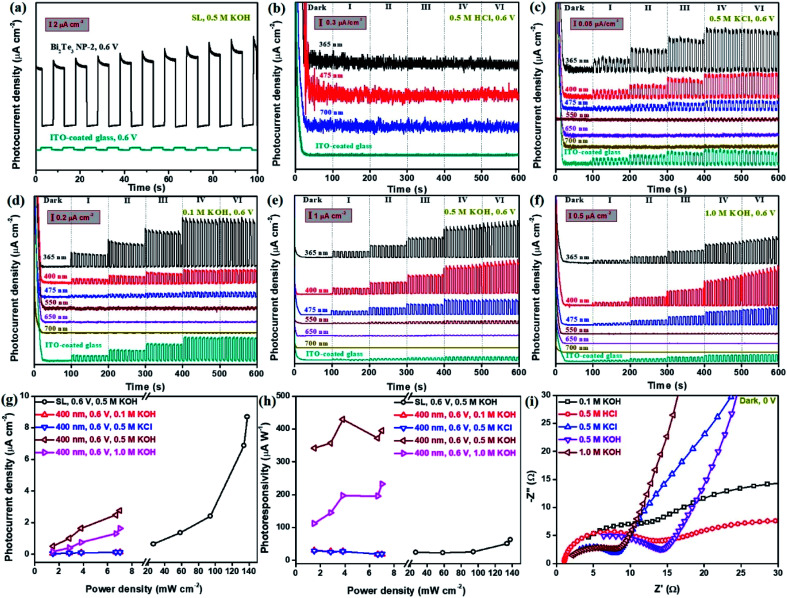
The photoresponse behaviours of Bi_2_Te_3_ NP-2-based photodetectors in various electrolytes under a SL and light with various wavelengths. (a) The on/off switching behaviours triggered by a SL in 0.5 M KOH at 0.6 V at 134 mW cm^−2^. (b–f) The on/off switching behaviours in various electrolytes under light with wavelengths of 365, 400, 475, 550, 650, and 700 nm at 0.6 V in (b) 0.5 M HCl, (c) 0.5 M KCl, (d) 0.1 M KOH, (e) 0.5 M KOH, and (f) 1.0 M KOH. For clarity, the photoresponse profile of naked ITO-coated glass irradiated by using a SL was added. (g) *P*_ph_ as a function of light power density under a SL and light with various wavelengths. (h) *R*_ph_ as a function of light power density under a SL and irradiation with various wavelengths. (i) EIS patterns of Bi_2_Te_3_ NPs-2 in various electrolytes under dark environments at 0.6 V.

The typical photoresponse behaviour of the Bi_2_Te_3_ NP-based photodetector was evaluated using a PEC system equipped with a standard three-electrode configuration, as shown in Scheme S1.†Fig. 3a gives the patterns of the as-fabricated Bi_2_Te_3_ NPs cast onto ITO-coated glass, exhibiting strong on/off switching photoresponse behaviours at an applied bias voltage of 0.6 V. For clarity, the profile of the naked ITO-coated glass was added in Fig. 3a, which displays a negligible signal as compared to Bi_2_Te_3_ NPs under the same conditions, revealing that the photoresponse signal indeed comes from the Bi_2_Te_3_ NPs rather than ITO. In addition, it should be noted that the type of electrolytes plays an important role in the PEC performance. As is shown in Fig. 3b, c and e, Bi_2_Te_3_ NPs-2 irradiated by using several single-wavelength lasers, exhibit an excellent on/off switching photoresponse behaviour in 0.5 M KCl (Fig. 3c) and 0.5 M KOH (Fig. 3e) while they show poor performance in 0.5 M HCl (Fig. 3b) at 0.6 V, suggesting that KCl and KOH are preferred electrolytes for Bi_2_Te_3_ NPs in this PEC system but HCl is not. Besides, the influences of lasers with different *λ* and *P*_*λ*_ on the PEC performance were investigated (Fig. 3c–f). Six lasers with specific *λ* (*λ* = 365, 400, 475, 550, 650, and 700 nm) and *P*_*λ*_ were employed to demonstrate the laser wavelength-dependent PEC performance of Bi_2_Te_3_ NPs. Similarly, the profiles of naked ITO-coated glass irradiated by using a SL are also added for comparison in Fig. 3c–f. It can be observed that when the *λ* value is less than 550, Bi_2_Te_3_ NPs show a strong PEC signal in KCl and KOH and the signal gradually increases with the decrease of the *λ* value (Fig. 3c–f), which can be attributed to the relatively highe laser energy and *P*_*λ*_ (Table S1†). However, when *λ* ≥ 550 nm, a negligible photoresponse signal of Bi_2_Te_3_ NPs can be observed, different from the results in absorption spectra, which is attributed to the very weak laser energy employed for PEC measurements in this work. In addition, it should be pointed out that the signal of naked ITO-coated glass irradiated by using a SL with much higher *P*_*λ*_ is obviously lower than that of Bi_2_Te_3_ NPs (Fig. 3c–f) irradiated by using single-wavelength lasers with shorter *λ*, *e.g.*, 365 nm, while higher than that irradiated by using single-wavelength lasers with longer *λ*, *e.g.*, 650 nm and 700 nm, reconfirming the truth of the PEC signal of Bi_2_Te_3_ NPs. Furthermore, it shows the same trend when the *P*_*λ*_ gradually increases from dark to VI in both KCl and KOH, that is, the PEC signal of Bi_2_Te_3_ NPs increases with the *P*_*λ*_ (Fig. 3c–f). To quantitatively evaluate the photoresponse performance of Bi_2_Te_3_ NPs, the photocurrent density (*P*_ph_) and photoresponsivity (*R*_ph_) can be obtained by:^43,44^

1 *P*_ph_ = (*I*_light_ − *I*_dark_)/*S*

2 *R*_ph_ = *P*_ph_/*P*_*λ*_

where, *I*_light_ and *I*_dark_ are the drain current with and without light, respectively; *P*_*λ*_ and *S* are the light power density and effective area of the Bi_2_Te_3_ NPs on ITO-coated glass, respectively. The bias voltage dependent on the PEC performance was also studied (Fig. 3e and S1†). The *P*_ph_ of Bi_2_Te_3_ NPs-2 in 0.5 M KOH gradually increases with the applied bias voltage, *i.e.*, the *P*_ph_ of Bi_2_Te_3_ NPs-2 irradiated by using a 365 nm laser increases from 44.8 nA cm^−2^ (0 V), to 96.4 nA cm^−2^ (0.3 V), to 2.52 μA cm^−2^ (0.6 V), similar to that in 0.5 M KCl (Fig. 3c and S2†). Photocurrent generation of the Bi_2_Te_3_ NPs at 0 V means that the Bi_2_Te_3_ NP-based photodetector is able to display self-powered PEC performance in KOH, yet additional external bias voltage can strengthen the photocurrent generation, which can be ascribed to the fact that the external bias voltage across the photoelectrode can construct a potential gradient within Bi_2_Te_3_ NPs and enhance the separation of photogenerated holes and electrons.^45^ Therefore, we think that the photoresponse mechanism of Bi_2_Te_3_ NPs is similar to that of bismuthene and tellurene: (i) formation of electron (e^−^)–hole (h^+^) pairs by photoexcitation and (ii) photoinduced charge transportation.^10,11^ Surprisingly, it should be noted that the *P*_ph_ and *R*_ph_ of Bi_2_Te_3_ NPs can reach up to 8.68 μA cm^−2^ and 395 μA W^−1^ (Fig. 3g and h), respectively, both of which largely outperform the reported bismuthene-based or tellurene-based photodetectors,^10,11^ which could be attributed to the unique property of the topological insulator, Bi_2_Te_3_. Notably, the *P*_ph_ of Bi_2_Te_3_ NPs in this work is also remarkably superior to those of ZnO homojunction nanowires (∼0.28 nA cm^−2^)^46^ and GaN nanowires (∼0.45 nA cm^−2^),^47^ considering the same effective area of the measured samples (2.2 cm^2^). Moreover, it is observed (Fig. 3g and h) that electrolyte concentration has a great effect on the PEC signal of Bi_2_Te_3_ NPs, that is, the *P*_ph_ and *R*_ph_ of Bi_2_Te_3_ NPs irradiated by using a 400 nm laser increase in the range of KOH concentration from 0.1 M to 0.5 M while both of them decrease from 0.5 M to 1.0 M, possibly ascribed to the slight electrochemical reaction at both bias voltage and high electrolyte concentration.^48^ The resistance (*R*) at the interface between the electrolyte and electrode gradually decreases with the increase of KOH concentration, *R*_0.1 M_ (16.3 Ω) > *R*_0.5 M_ (13.0 Ω) > *R*_1.0 M_ (8.09 Ω), while in 0.5 M electrolytes, the fact that *R*_HCl_ (13.3 Ω) > *R*_KOH_ (13.0 Ω) > *R*_KCl_ (7.25 Ω) (Fig. 3i) can be due to the different functionalities between Bi_2_Te_3_ NPs and electrolytes, similar to the reported results of black phosphorus nanosheets^49^ and bismuth sulfide(iii) nanosheets.^45^

**Fig. 4:**
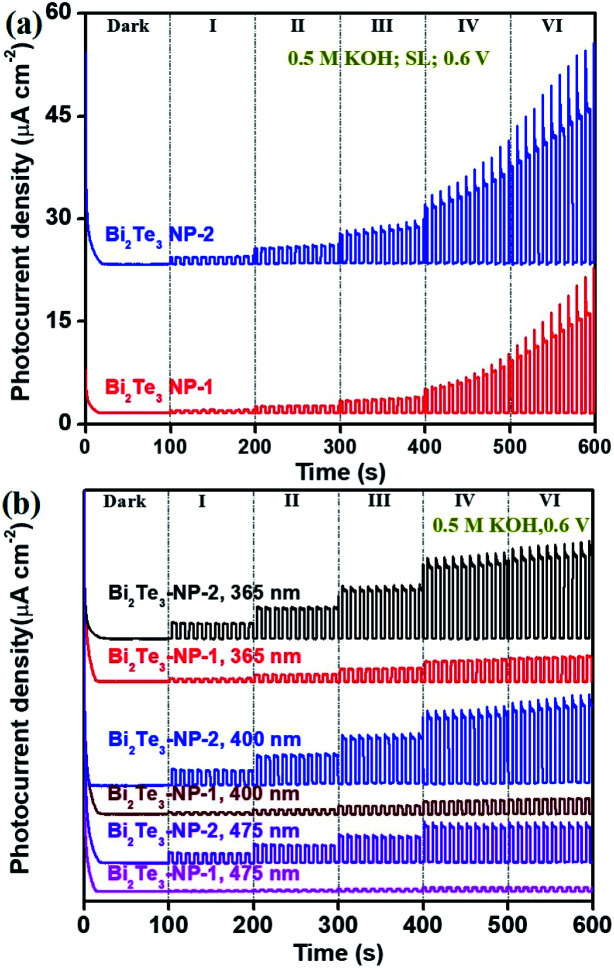
Size effect of Bi_2_Te_3_ NPs on the photoresponse performance in 0.5 M KOH under (a) a SL and (b) three different lasers (365, 400, and 475 nm).

Due to the size-dependent *E*_g_ of Bi_2_Te_3_ NPs (Fig. 2g), the photoresponse behaviours of the Bi_2_Te_3_ NPs-1 and Bi_2_Te_3_ NPs-2 irradiated by using a SL and three kinds of lasers (365, 400, and 475 nm) were studied to understand the influence of the size of Bi_2_Te_3_ NPs on the PEC performance, as shown in Fig. 4. It can be seen that the PEC signals of both Bi_2_Te_3_ NPs-1 and Bi_2_Te_3_ NPs-2 increase with the *P*_*λ*_ (Fig. 4a) and show the same trend as those of the Bi_2_Te_3_ NPs-2, *i.e.*, the PEC signal declines as the *λ* value increases (Fig. 4b). In addition, it is noted that the PEC signal of Bi_2_Te_3_ NPs-2 is obviously stronger than that of Bi_2_Te_3_ NPs-1, no matter which laser was employed (Fig. 4a and b), *e.g.*, the *P*_ph_ of Bi_2_Te_3_ NPs-2 irradiated by using a 400 nm laser at 4.65 mW cm^−2^ is 2.52 μA cm^−2^ while that of Bi_2_Te_3_ NPs-1 is only 0.729 μA cm^−2^. This could be attributed to the synergistic effect of the suitable *E*_g_ and the number of accessible active sites on the Bi_2_Te_3_ NPs-2. Since the *E*_g_ of Bi_2_Te_3_ NPs inversely correlates with size, the larger Bi_2_Te_3_ NPs-1 have stronger absorption under incident light. However, with the size decrease of Bi_2_Te_3_ NPs, the specific surface area becomes higher and accessible active sites on the PEC performance of Bi_2_Te_3_ NP-based photodetectors, similar to previously reported results.^10,44,50^ Therefore, the size of nanomaterials has a great influence on the PEC performance; Bi_2_Te_3_ NPs tend to be larger, resulting in higher efficiency, which provides fundamental acknowledgement of the design and optimization of PEC-type devices.

**Fig. 5:**
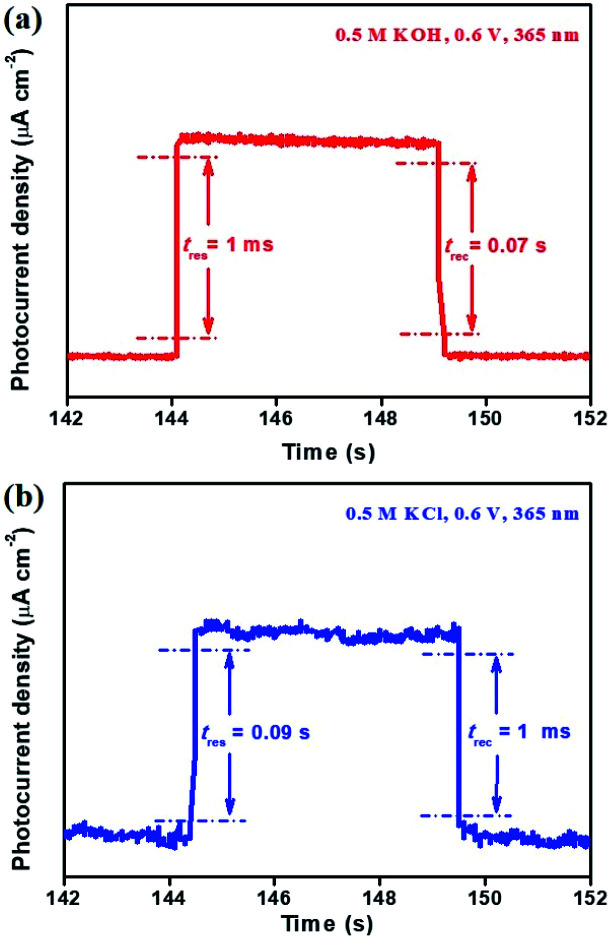
The *t*_res_ and *t*_rec_ of (a) Bi_2_Te_3_ NPs-1 in 0.5 M KOH and (b) Bi_2_Te_3_ NPs-2 in 0.5 M KCl, irradiated by using a 365 nm laser.

In addition, the response time (*t*_res_) and recovery time (*t*_rec_) of the Bi_2_Te_3_ NP-based photodetector were ascribed to the time interval for the rise and decay from 10% to 90% and from 90% to 10% of its peak value, respectively.^48,51^ It can be observed that regardless of the size of Bi_2_Te_3_ NPs and the type of electrolytes, the Bi_2_Te_3_ NP-based photodetector always shows fast *t*_res_ (0.001–0.09 s) as well as *t*_rec_ (0.001–0.07 s) (Fig. 5), both of which are superior to those of the bismuthene-based photodetector (*t*_res_ = 0.2 s, *t*_rec_ = 0.2 s),^11^ tellurene-based photodetector (*t*_res_ = 0.2 s, *t*_rec_ = 0.2 s),^10^ ZnO homojunction nanowires (*t*_res_ = ∼50 s, *t*_rec_ = ∼200 s)^46^ and GaN nanowires (*t*_res_ = 0.003 s, *t*_rec_ = 0.003 s).^47^ This could be attributed to the unconventional phase of Bi_2_Te_3_ quantum matter. This indicates that the Bi_2_Te_3_ NP-based photodetector has appealing potential in the field of PEC-type devices.

**Fig. 6:**
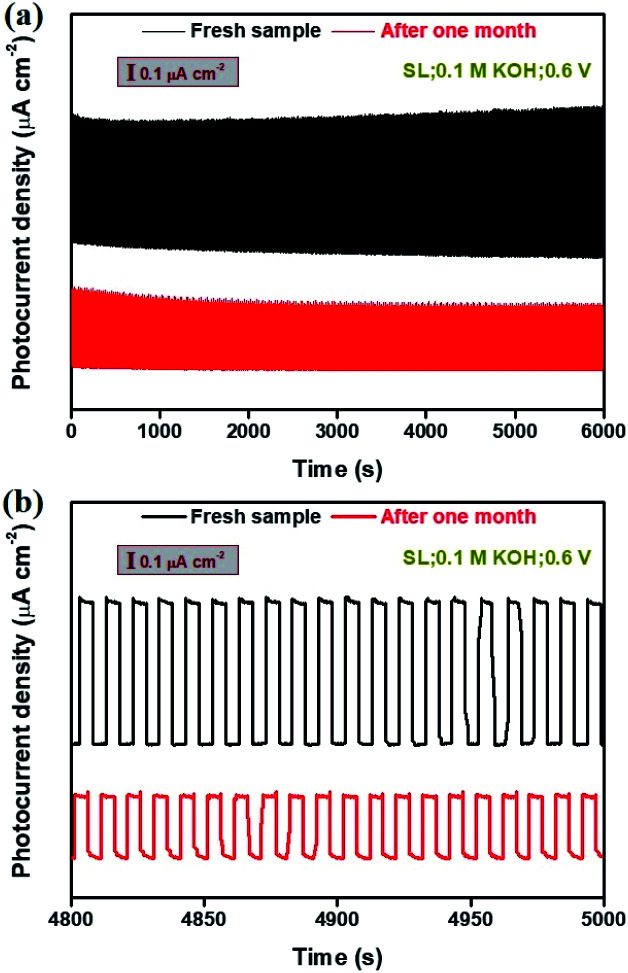
Long-term stability of the photoresponse performance of the Bi_2_Te_3_ NPs-1 under a SL in 0.1 M KOH (a) before and (b) after one month.

Long-term stability measurements of the photoresponse of Bi_2_Te_3_ NP-based photodetectors are of great importance for practical application. Fig. 6 gives the stability profiles of the Bi_2_Te_3_ NP-1-based photodetector irradiated by using a SL in 0.1 M KOH at a bias voltage of 0.6 V. The 600 cycles with 5 s intervals of on/off switching were traced (Fig. 6a), and the 481–500^th^ cycles were chosen to evaluate the PEC stability of the Bi_2_Te_3_ NP-1-based photodetector (Fig. 6b). It should be pointed out that no obvious change is observed by visual inspection of Bi_2_Te_3_ NPs-1 specimens after PEC stability measurements in 0.1 M KOH (Fig. S3†), indicating the excellent PEC stability of the as-fabricated Bi_2_Te_3_ NP-1-based photodetector. A notable on/off switching behaviour can be observed even after one month, suggesting the long-term PEC stability of Bi_2_Te_3_ NPs under ambient conditions. Furthermore, it is calculated from Fig. 6b that the *P*_ph_s of the fresh Bi_2_Te_3_ NPs-1 in the 481–500^th^ cycles is 879 nA cm^−2^ in 0.1 M KOH, declining to 439 nA cm^−2^ after one month. An approximate reduction of 50.1% of *P*_ph_ was obtained, comparable to that of the bismuthene-based photodetector^11^ and tellurene-based photodetector.^10^ The decline of *P*_ph_ could be ascribed to the weak electrochemical reaction at high bias voltage (0.6 V) under a SL with high *P*_*λ*_ (134 mW cm^−2^) and slight peel-off during long-running measurements, which can be efficiently solved by coating conductive polymers, such as polyaniline and polypyrrole, onto the surface of Bi_2_Te_3_ NPs to remarkably lower the electrochemical reaction of Bi_2_Te_3_ NPs and employ stronger binders to make Bi_2_Te_3_ NPs more strongly fixed on the surface of ITO-coated glass to avoid the slight peel-off during long-running measurements.

## Conclusions

In summary, topological insulator Bi_2_Te_3_ NPs were successfully synthesized by a solvothermal approach and the size of Bi_2_Te_3_ NPs can be readily controlled by simply tuning the reaction time. The hexagonal structure of Bi_2_Te_3_ NPs was well-characterized, and UV-Vis spectra revealed a broadband absorption range from 260 nm to 1500 nm. The as-synthesized Bi_2_Te_3_ NPs were, for the first time, employed as a working material in a PEC-type photodetector. The PEC result not only shows that the *P*_ph_ and *R*_ph_ significantly improve but also exhibits faster *t*_res_ and *t*_rec_, compared to those of bismuthene-based or tellurene-based photodetectors. It was also shown that Bi_2_Te_3_ NPs-2 displayed better PEC performance, attributed to the synergistic effect of optical absorbance and the number of accessible active sites on a Bi_2_Te_3_ NP. In addition, good PEC stability of the Bi_2_Te_3_ NP-based photodetector was obtained in 0.1 M KOH after one month without any protection, comparable to the bismuthene-based or tellurene-based photodetector. Because of the facile synthesis, easy size control of Bi_2_Te_3_ NPs, excellent photoresponse performance, and good long-term stability of the Bi_2_Te_3_ NP-based photodetector, we believe that Bi_2_Te_3_ can pave a new way for the design of bismuthene or tellurene nanomaterial-based high-performance PEC-type devices with practical applicability.

## Conflicts of interest

There are no conflicts to declare.

## Supplementary Material

NA-002-D0NA00006J-s001
