# Supplementary material for: Few-layer hexagonal bismuth telluride (Bi2Te3) nanoplates with high-performance UV-Vis photodetection
Source: Nanoscale Adv. 2020 Feb 10;2(3):1333–9. doi: 10.1039/d0na00006j (PMC9419258; doi:10.1039/d0na00006j)
Supplement: NA-002-D0NA00006J-s001 [file NA-002-D0NA00006J-s001.pdf]

Electronic Supplementary Material (ESI) for Nanoscale Advances.
This journal is © The Royal Society of Chemistry 2020

Supporting information for

Few-Layer Hexagonal Bismuth Telluride (Bi_2Te_3) Nanoplates with High-Performance UV-Vis Photodetection

Ye Zhang,‡[a] Qi You,‡[a] Weichun Huang,*[b] Lanping Hu,[b] Jianfeng Ju,[b] Yanqi Ge,*[a] and Han Zhang*[a]

Table S1 Light powder density (P_λ) of simulated light (SL) and single-wavelength lasers. The gradually increased P_λ were labelled with I, II, III, IV, and VI levels, respectively.

P_λ (mW cm^{-2})	I Level	II Level	III Level	IV Level	VI Level
SL	26.6	58.3	94.2	134	138
365 nm	0.920	2.17	3.11	4.42	4.65
400 nm	1.49	2.84	3.82	6.67	7.01
475 nm	2.31	4.87	7.26	11.0	11.5
550 nm	1.81	3.82	6.05	8.37	8.64
600 nm	2.13	4.24	5.81	9.59	9.76
650 nm	1.95	4.11	6.43	9.15	9.43
700 nm	1.34	2.68	4.23	5.99	6.18

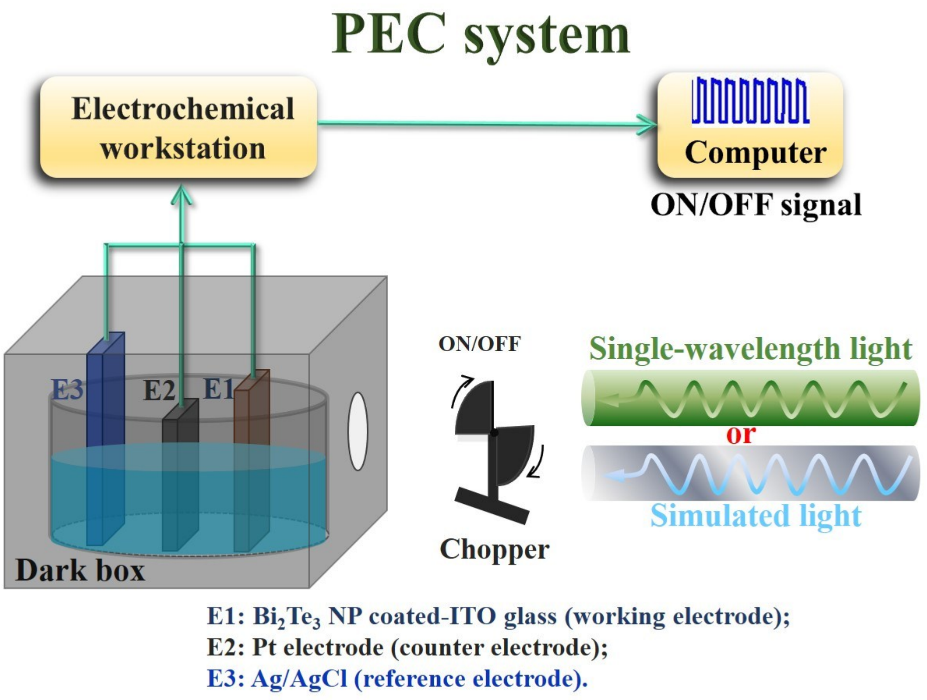

Scheme S1. A typical photoelectrochemical system built for evaluating the photoresponse behaviour of the Bi_2Te_3 NP-based photodetector in electrolytes.

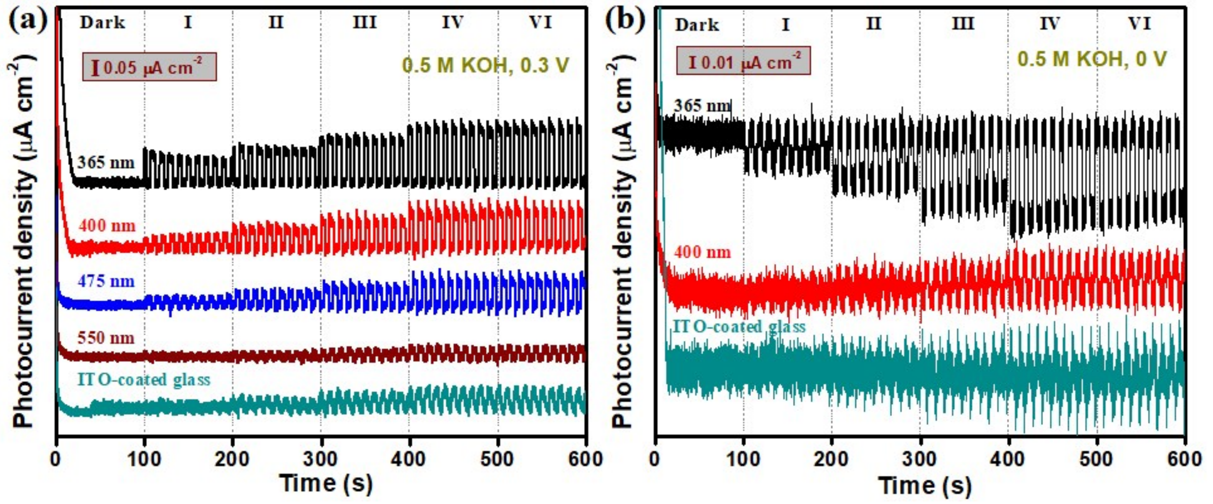

Fig. S1. The photoresponse behaviours of the Bi_2Te_3 NPs-2-based photodetectors under lasers with different wavelengths in 0.5 M KOH at bias voltages of (a) 0.3 V and (b) 0 V. For clarity, an ITO-coated glass irradiated by a SL was added.

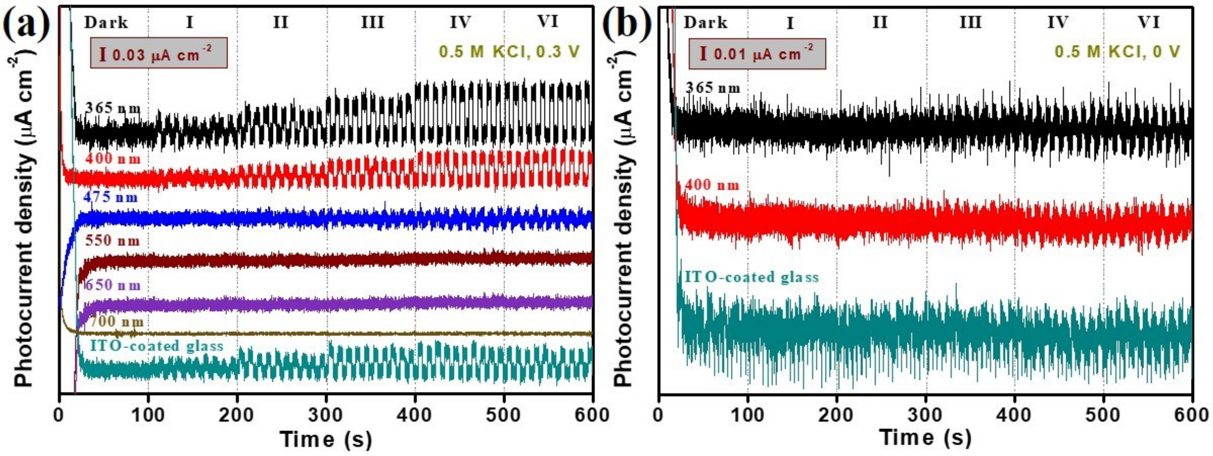

Fig. S2. The photoresponse behaviours of the Bi_2Te_3 NPs-2-based photodetectors under lasers with different wavelengths in 0.5 M KCl at bias voltages of (a) 0.3 V and (b) 0 V. For clarity, an ITO-coated glass irradiated by a SL was added.

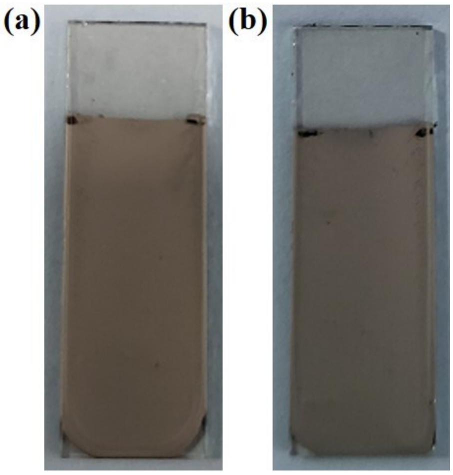

Fig. S3. Photographs of 2D Bi_2Te_3 NPs-1 on the ITO-coated glass before and after one-month PEC stability measurement.
